# Clasificando mujeres: diagnósticos psiquiátricos y subjetividad femenina en el Manicomio Provincial de Málaga, España, 1909-1950

**DOI:** 10.1590/S0104-59702023000100003

**Published:** 2023-04-03

**Authors:** Celia García-Díaz, Isabel Jiménez-Lucena

**Affiliations:** i Profesora asociada área Historia de la Ciencia/Universidad de Málaga. Málaga – Andaluzia – España celiagarciad@uma.es; ii Profesora titular área Historia de la Ciencia/Universidad de Málaga. Málaga – Andaluzia – España isajimenez@uma.es

**Keywords:** género, historia de la psiquiatría, mujeres, Manicomio Provincial de Málaga, gender, history of psychiatry, women, Manicomio Provincial de Málaga

## Abstract

Las historias clínicas de manicomios de mujeres permiten ahondar en la brecha que se abre entre la ilusión positivista de la psiquiatría durante la primera mitad del siglo XX en España y la vivencia subjetiva del internamiento psiquiátrico de las mujeres-locas doblemente subalternas. Las clasificaciones diagnósticas fueron claves en este intento de positivización. El objetivo de este trabajo es señalar qué elementos subjetivantes participaron en la aplicación de diagnósticos como esquizofrenia, psicopatía y oligofrenia en la sala de mujeres del Manicomio Provincial de Málaga, y mostrar cómo el ideal hegemónico de feminidad estableció un límite permeable entre la cordura y la locura de las mujeres, entre asimilaciones y resistencias.

## Abstract (en)

The clinical histories of women’s asylums allow us to deepen the gap between the positivist illusion of psychiatry during the first half of the 20th century in Spain and the subjective experience of the psychiatric internment of doubly subaltern crazy women. Diagnostic classifications were key in this attempt at positivization. This paper aims to point out which subjectifying elements participated in the application of diagnoses such as schizophrenia, psychopathy, and oligophrenia in the women’s wards of the Manicomio Provincial de Málaga, and to show how the hegemonic ideal of femininity established a permeable limit between sanity and madness of women, between assimilations and resistances.

Es bien conocido que a partir de los años 1960 las aportaciones de la escuela constructivista tuvieron una remarcable importancia al poner en evidencia el carácter social de la producción científica, abriendo nuevos campos de estudio (Jiménez-Lucena, Ruiz Somavilla, 1999, p.81). Obras como *Historia de la locura* (Foucault, 1972) e *Historia de la sexualidad* (Foucault, 2006) favorecieron el estudio de la construcción social de la locura a la vez que, en el movimiento anti psiquiátrico, autores como Thomas Szasz y Erving Goffman afirmaban que la enfermedad mental no debía entenderse como un hecho natural sino como un constructo cultural sustentado por una red de prácticas administrativas y clínico-asistenciales (Porter, 2002, p.15). La psiquiatría, como especialidad médica, se fundamentó en la legitimación de estrategias biopolíticas, desarrollando la normalización-patologización de conductas, actitudes, ideas, cuerpos y formas de vida, creando nuevas fronteras cambiantes, según los contextos, entre lo normal y lo anormal. Durante el siglo XX, la psiquiatría se desarrolló como un dispositivo de control biopolítico, alejándose cada vez más de los delirios y la locura, y actuando sobre cualquier tipo de conducta, con fines reguladores, como indicó Foucault en sus escritos (Márquez Estrada, 2014). Por otro lado, se estaban poniendo en evidencia las múltiples tensiones cognitivas, emocionales y sociales a las que se encuentra sometido el llamado “sistema género-ciencia” frente a una idea de inalterabilidad del sistema que era clave para perpetuar el contexto de dominación en relación con el sexo-género (Sánchez, 2003, p.14). La categoría de análisis “género” puso de manifiesto el papel que el sexo, como dispositivo biopolítico, o dispositivo de poder que regulaba (y regula) las vidas y los cuerpos, controlaba (y controla) las sexualidades, conformando ideas hegemónicas de sexo binario, y patologizando sexualidades disidentes. El cuerpo-mente de las mujeres ha sido psiquiatrizado en ese afán biopolítico regulatorio, produciendo subjetividades que han generado respuestas de las mujeres que han ido de la asimilación al rechazo y la resistencia.

En este contexto las investigadoras feministas que se habían interesado por la construcción social de la locura analizaron qué había ocurrido con las mujeres en hospitales y gabinetes psiquiátricos, cómo habían sido diagnosticadas y qué elementos habían favorecido la “feminización de la locura” (Chesler, 1972; Showalter, 1985). En la modernidad europea, se produjo un cambio en la visión de la regulación social pasando de estar en manos de la religión a ser la ciencia la encargada de ejercer dicho papel. Una actividad científica influida y conformada por elementos extra epistémicos como una nueva concepción de individuo, reflexividad del yo, conflictos entre lo público y lo privado y ruptura de tradiciones, entre otros factores (Novella, 2018). Este cambio favoreció un discurso en torno al sexo y a su papel social basado en teorías científicas que se ocupó de transmitir un *statu quo* para las mujeres; esto ocurrió de forma más evidente durante el final del siglo XIX y principios del XX, en un intento de frenar los cambios sociales que el movimiento feminista planteaba.^1^ En el seno de la comunidad alienista, como precursora de la especialidad psiquiátrica, se construyeron argumentos sobre la inferioridad intelectual de las mujeres que permitieron la elaboración de etiquetas diagnósticas con fines reguladores del rol social femenino (Sowerwine, 2003).

En este sentido no cabe pensar en una ciencia portadora de una objetividad absoluta que define una realidad universal y única, y hay que considerar la relación de conocimientos y prácticas con la legitimación y mantenimiento de clichés y sesgos que, manejados de forma no reflexiva, han creado estructuras y compartimentos rígidos donde se clasifican los comportamientos de las mujeres desde una lógica de contrarios (Ruiz Somavilla, Jiménez-Lucena, 2003; Jiménez-Lucena, 2014). En la búsqueda de una fundamentación positiva de la patología psiquiátrica, los órganos sexuales femeninos, con su capacidad para producir excitaciones eróticas, fueron considerados el sustrato físico de las alteraciones del comportamiento de las mujeres y, por tanto, el origen de las enfermedades nerviosas femeninas (Jordanova, 1989; Moscucci, 1993; Lupton, 1994; Tomes, 1994). Por otro lado, el desarrollo de la endocrinología también influyó en la construcción generizada de la locura. Durante los años 1920, el estudio de las hormonas se desarrolló en el ámbito anglosajón (Inglaterra y EEUU) en un intento de aislar sustancias químicas que dieran forma a los cuerpos y determinaran su sexualidad. Se pensaba que las hormonas sexuales estaban directamente relacionadas con el desarrollo de enfermedades mentales por lo que el estudio de tejido hormonal que procedía de pacientes psiquiátricos fue, durante varias décadas, objeto de investigación en un intento de buscar conexiones entre lo mental y lo hormonal (Hirshbein, 2010, p.163; Evans, Jones, 2012). En el caso de las mujeres, las relaciones que se establecieron entre las hormonas y la mente (y el cerebro) aportaron justificaciones científicas para patologizar diferentes etapas de la biología femenina como la menarquia, la menopausia o los periodos postpartos.

En un contexto histórico social de cambio con un creciente movimiento feminista fue crucial establecer criterios, medidas y etiquetas que definieran una feminidad sana, así como las líneas que separaban a la “mujer cuerda” de la “mujer loca”. Lo que ha sido deseable en diferentes momentos, las necesidades del sistema de producción y reproducción han provocado modificaciones de las definiciones del ideal de “mujer” con un objetivo regulador, generándose discursos sobre la feminidad en los que la incipiente ciencia psiquiátrica jugó un papel protagonista (Chesler, 1972).

El objetivo de nuestro trabajo es mostrar cómo los diagnósticos psiquiátricos se ajustaron a los contextos sociales y políticos con la finalidad de configurar subjetividades que legitimaran y perpetuaran un orden sociopolítico determinado estrechamente relacionado con la feminidad hegemónica. El manicomio, como espacio donde se genera un juego de subjetividades, es el escenario de nuestra investigación que trata de hacer perceptibles mecanismos de dominación y explotación, evidenciando cómo el discurso psiquiátrico no escapó del contexto político en el que fue generado. Para ello analizaremos los elementos que influyeron en la construcción de la subjetividad de las mujeres-locas desde lo local/global, centrándonos en la sala 20 del Manicomio Provincial de Málaga durante el periodo comprendido entre 1909 y 1950.^2^ Las fuentes del estudio han sido 811 historias clínicas de mujeres ingresadas en esa institución durante los años estudiados, conservadas en el Archivo de la Diputación Provincial de Málaga (ADPM) y Fondo Pedro Ortiz Ramos del Archivo Universitario de Granada. Los documentos han sido analizados desde una doble metodología cuantitativa y cualitativa.^3^ Estas fuentes no solo aportan datos en torno a las prácticas asistenciales, sino que también nos hablan de contextos culturales y políticos, así como de los procesos de regulación social que en ellos han tenido lugar (Moliniari, 2005). En este sentido, conocer los cambios en el ideal femenino de la época es central para la contextualización y la interpretación de las fuentes historiográficas. Su influencia en la elaboración de un conocimiento psiquiátrico en torno a la biología femenina planteará claves sobre la regulación de las psiques, los cuerpos y, en definitiva, la vida de las mujeres.

## Psiquiatría y cambio de la feminidad en la primera mitad del siglo XX en España

A partir de la segunda mitad del siglo XIX en España se produjeron cambios en el ideal femenino si bien el movimiento feminista como movimiento social encontró limitaciones como el alto nivel de analfabetismo, el clima político conservador y el protagonismo de la moral católica (Nash, 1994). Estos cambios estuvieron sujetos a fuerzas de distinto signo: por un lado, la introducción del positivismo científico, así como el deseo de perpetuar un orden social liberal-capitalista enaltecieron la idea de la mujer tradicional; por otro, el Krausismo,^4^ el empuje de la lucha de las mujeres obreras por conquistar derechos sociales en las fábricas y, posteriormente, el acceso de las mujeres a la política durante la Segunda República, fundamentaron el empoderamiento de las mismas, haciendo posible una visión de la feminidad que incluía la lucha activa, el acceso a la cultura y al trabajo asalariado, así como cambios en las políticas sobre la maternidad y la anticoncepción introducidas, en gran medida, a través de revistas del ámbito libertario (Jiménez-Lucena, Molero-Mesa, Tabernero-Holgado, 2013; Molero-Mesa, Jiménez-Lucena, Tabernero-Holgado, 2018). De esta forma, durante los años 1920 y 1930 el ideal sobre la feminidad alcanzó cambios que desafiaron los límites entre los sexos y las mujeres comenzaron a hacerse visibles en los espacios reservados a los hombres como los estudios superiores y puestos de relevancia política.

En esta dinámica, desde el ámbito científico se elaboró un discurso que trataba de resistir el cambio del papel de las mujeres en la sociedad, esencializando los sexos biológicos y configurando una división sexualizada del trabajo. La frenología aportó estudios fuertemente ideologizados, tanto antropométricos como de las circunvoluciones cerebrales y medidas craneales que fueron usados para justificar la construcción de la inferioridad mental de la mujer reforzando un discurso médico que sujetaba a las mujeres al rol tradicional (Bosch Fiol, Ferrer Pérez, 2003). En España, Juan Giné y Partagás, médico alienista barcelonés, aseguraba en 1882 que la mujer tenía mayor predisposición a la enfermedad mental y que todo lo relacionado con la domesticidad y el cuidado de la casa y la familia ejercía un papel protector sobre la locura que la acechaba de manera natural (Diéguez Gómez, 1999, p.643). Ya entrado el siglo XX, Roberto Novoa Santos (1885-1933) defendió la inferioridad mental de la mujer en España, asegurando: “Resulta pues que, de cien mujeres originales, las cien son degeneradas, sujetos que caen dentro del terreno de la psicopatología” (Novoa Santos, 1908, p.116).^5^ Por tanto, en la elaboración de la división sexual del trabajo jugaron un papel importante la consulta psiquiátrica y el dispositivo manicomial.

A finales del siglo XIX y principios del XX, creció el interés de los médicos españoles en afianzar la relación entre el aparato reproductor femenino y el sistema nervioso (Jiménez-Lucena, Ruiz Somavilla, 1999, p.195). Esto llegó a generar diferencias terapéuticas; así, Arturo Galcerán, médico de la España de la época, trató en 1883 las monomanías impulsivas de forma diferente según los pacientes: si eran hombres, les aplicaba morfina e hidroterapia; si era mujer, el tratamiento se centraba en el aparato genital, con inyecciones vaginales de agua tibia, cauterizaciones con nitrato de plata o aplicación de tintura de yodo en el cuello del útero y vagina (Diéguez Gómez, 1999, p.646). En la *Revista Frenopática Española*,^6^ durante la primera década del siglo XX, las publicaciones sobre la intervención ginecológica como tratamiento de desórdenes mentales estaban en auge, mediante la exposición de casos concretos de curación de melancolías y crisis epilépticas al extirpar un quiste ovárico o de remisión de psicosis puerperales al intervenir sobre el aparato genital femenino. Sin embargo, en los países pioneros en estas prácticas, a finales del siglo XIX, habían comenzado a denunciarse por poco efectivas, dejando paso a nuevas terapéuticas para inmovilizar a las mujeres como el uso de la cocaína o el láudano para los llamados estados premenstruales (Jiménez-Lucena, Ruiz Somavilla, 1999, p.200-201).^7^

En los años 1920 y 1930 se desarrollaron nuevas ideas sobre la feminidad asociadas tanto al movimiento feminista burgués, centrado en la reivindicación del sufragismo, como al ámbito obrero y libertario en el que destacaron las propuestas centradas en la igualdad real entre hombres y mujeres, la crítica al matrimonio como institución de sometimiento y el desarrollo de planteamientos rupturistas de las ideas tradicionales de la pareja y del amor (Vicente, 2014). Durante la Segunda República Española, las reivindicaciones de las mujeres cristalizaron en la redacción de leyes concretas que supusieron grandes avances sociales como la implementación del permiso por maternidad, la legalización del voto femenino y la mayor visibilización en puestos políticos, así como el auge de un feminismo organizado dentro del anarquismo (Sánchez Blanco, 2007). Estas nuevas ideas tuvieron diferentes respuestas en la comunidad científica en la que se estableció una dinámica ambivalente de aceptación y rechazo. Uno de los más eminentes médicos españoles de la primera mitad del siglo XX, Gregorio Marañón, construyó un discurso en torno a la construcción de la feminidad y la masculinidad que generó una gran controversia en el feminismo de la época. Por un lado, este médico se afanó en ensalzar la diferencia sexual en los aspectos reproductivos, señalando el papel fundamental de la maternidad como eje central de su discurso en torno a la biología femenina. En su obra *Sexo, trabajo y deporte*, publicada en 1926, señalaba que la diferencia entre los sexos no solo estaba inscrita en los cuerpos, a nivel anatómico, sino que esa anatomía propiciaba una diferenciación funcional: mientras los hombres eran más aptos para el trabajo por su amplitud torácica, el cuerpo de las mujeres estaba orientado a la procreación, por el mayor tamaño de sus órganos reproductivos (Castejón Bolea, 2013). Planteaba así su rechazo a un “feminismo virilizante” que aspiraba a asumir los mismos privilegios que los hombres. Por otro lado, sus teorías fueron fuertemente criticadas por una parte del feminismo español que defendía un feminismo igualitario. En este sentido, la crítica estaba centrada en dos aspectos: el primero, mostraba que la teoría de Marañón seguía estando basada en la inferioridad de la mujer, suponiendo que la feminidad se situaba entre la adolescencia y la madurez viril; el segundo, criticaba esa separación entre femenino, masculino y hermafrodita que planteó en algunos trabajos. María Cambrils^8^ rechazaba la educación maternal en las mujeres ya que reforzaba el papel central de la procreación y planteó un concepto de individuo por encima de la categoría sexual, desde un feminismo más igualitario. Ella argumentaba que los hombres sensibles y las mujeres viriles no eran aberraciones de la naturaleza, sino formas indiferenciadas que habían alcanzado un mayor grado de perfeccionamiento desde una perspectiva evolucionista (Aresti, 2001, p.248). En este marco, la introducción del psicoanálisis en la medicina española también jugó un papel fundamental en estas dinámicas ambivalentes.^9^ Algunos psiquiatras como César Juarros,^10^ Sanchis Banús y Ángel Garma, señalaron una relación directa entre la represión sexual en las mujeres y el padecimiento de trastornos mentales (Huertas, Novella, 2013), mientras defendían la necesidad de una educación sexual desde la infancia, para niños y adolescentes, sin mencionar a niñas y mujeres, volviendo a poner en manos de los hombres la sexualidad femenina.^11^ Con este giro discursivo se reforzaba el papel de los órganos sexuales femeninos y su fisiología con la etiología de la enfermedad mental en las mujeres.

Los avances en los derechos de las mujeres que tuvieron lugar durante la Segunda República se vieron interrumpidos bruscamente con el estallido de la Guerra Civil. Las mujeres participaron activamente en la contienda asumiendo diferentes roles. En Málaga colaboraron en actividades sindicales, pertenecían a comités de trabajo y apoyaron la prensa local republicana. También las mujeres milicianas participaron en la vanguardia, con una conciencia política muy desarrollada (Barranquero Texeira, Eiroa San Francisco, 2011, p.127).

El primer franquismo desplegó una serie de estrategias para legitimar el nuevo régimen político implantado por la fuerza. Entre estas estrategias, el régimen franquista se aseguró de eliminar o invisibilizar los fundamentos y las prácticas de la reforma moral y sexual de la Segunda República. La regulación de la moralidad de las mujeres fue un eje fundamental en la construcción de una “nueva raza” que pretendía una depuración ideológica, aunque como plantea Rosa Medina Doménech (2013), no todas las mujeres se ajustaron a esta idea de la feminidad nacional católica de forma pasiva, sino que desarrollaron estrategias para resistirse. Las mujeres fueron vistas como agentes que podían vehiculizar las ideas afines al régimen dentro del ámbito de lo doméstico, trasmitiendo estos valores a sus hijos e hijas. Así, incluso en los medios médico-sociales de la época, como la revista *Ser*, se llegó a plantear la reclusión de las mujeres solteras embarazadas en penitenciarios para estimular en ellas a través de la religión “el cariño al futuro hijo, inculcando un espíritu cristiano” (citado en Jiménez-Lucena, 1997, p.123). La vuelta a las concepciones decimonónicas sobre la idea de mujer y lo femenino, impregnó el régimen franquista, generalizándose la imagen de “ángel del hogar”, limitada al ámbito de lo doméstico y descrita con cualidades religiosas y místicas. Con este patrón, las mujeres rojas fueron el contra modelo, considerándolas responsables de la degeneración de la nación; por ello, se convirtieron en objetivo político.^12^ En este marco, se desarrolló una intensa patologización de la naturaleza femenina por parte de psiquiatras como Antonio Vallejo Nágera^13^ sosteniendo que:

Si la mujer es habitualmente de carácter apacible, dulce y bondadoso, débese a los frenos que obran sobre ella; pero como el psiquismo femenino tiene muchos puntos de contacto con el infantil y el animal, cuando desaparecen los frenos que contienen socialmente a la mujer y se liberan las inhibiciones frenatrices de las impulsiones instintivas, entonces despiértase en el sexo femenino el instinto de crueldad y rebasa todas las posibilidades imaginadas, precisamente por faltarle las inhibiciones inteligentes y lógicas (citado en Huertas, 1996, p.122).

Estas afirmaciones se relacionaban con la idea de que el marxismo encontraba en las personas degeneradas un caldo de cultivo perfecto, y, en especial, en las mujeres de esta ideología, asimiladas prácticamente con animales por el ideario franquista, de forma que ideario político marxista y psicopatía fueron estrechamente vinculados. En el ámbito de las prácticas de patologización de la resistencia a los regímenes políticos hegemónicos, las revoluciones y las protestas sociales, hay que considerar que las reivindicaciones feministas han supuesto una de las más relevantes interpelaciones a las sociedades modernas, por eso han llegado a ser un tema central para la psiquiatría como dispositivo biopolítico que ha jugado un papel clave en la regulación de las subjetividades. En la coyuntura histórica del franquismo la oposición de las mujeres al ideal femenino hegemónico y al régimen dictatorial establecido se constituyó en objetivo de las prácticas psiquiátricas.

## Entre la positivización y la subjetivación en el espacio manicomial de Málaga: esquizofrénicas, psicópatas y oligofrénicas

Los cambios sociales y económicos de la segunda mitad del siglo XIX y primera del XX coexistieron con la transformación del conocimiento psiquiátrico como autoridad que interpretaba conductas y argumentos como datos positivos, que ordenaba ingresos psiquiátricos y que podía llegar a autorizar la coerción en casos de rebeldía y resistencia al poder establecido con un interés legitimador de la joven medicina mental. El objetivo era mantener un orden sociopolítico determinado y para ello la locura vino a solventar el problema, transformándose en un manto bajo el cual cabían mujeres que trasgredían el orden establecido, por sus conductas, hábitos, rebeldía y resistencias (Jiménez-Lucena, Ruiz Somavilla, 1999, p.15; García-Díaz, Jiménez-Lucena, 2010). Las aportaciones de Erving Goffman (2009) sobre los mecanismos reguladores de la vida de los internados en instituciones totalitarias, entre ellas los manicomios, fueron centrales para el abordaje de las instituciones psiquiátricas desde la óptica del control. Entre estos elementos, el etiquetaje diagnóstico era necesario para clasificar conductas, hábitos, reacciones, emociones, e incluso indumentarias, e incluirlas en las clasificaciones diagnósticas psiquiátricas. Ian Hacking abordó, a finales de los 1990, las cuestiones clasificatorias y del etiquetaje diagnóstico como parte fundamental en el proceso de “construir/inventar personas”. Esos procedimientos podrían dar lugar a una asunción del diagnóstico por parte de los pacientes considerándolo un elemento identitario, o a su rechazo (Huertas, 2011). En el caso de las mujeres-locas, las justificaciones del etiquetaje diagnóstico podían llegar a ser extremadamente sutiles y subjetivas. El acto diagnóstico trataba de positivizar conductas consideradas “inadecuadas” o “inapropiadas” que las mujeres manifestaban en su entorno familiar y social, convirtiéndolas en síntomas y signos cuantificables, apelándose así a la intervención del discurso psiquiátrico y de la institución para la reconducción de la situación “anómala”.^14^ Los psiquiatras basaron su aproximación a la locura en el modelo kraepeliniano, que fue hegemónico en la psiquiatría occidental del siglo XX (Caponi, 2010). Según Caponi y Martínez Hernáez (2013, p.468) esta clasificación priorizó la positivización,^15^ minusvalorando los contenidos subjetivos de los discursos de los pacientes, creando lo que ellos describen como una “estrategia anti narrativa”, donde se oculta el discurso del paciente frente a las supuestas evidencias científicas que pretenden fundamentarse en una objetividad absoluta cuando tienen sus referentes en la subjetividad de quien diagnostica, clasifica. La historia clínica nos muestra una encrucijada discursiva donde la voz autorizada era la del experto, y se aprecia una cierta pérdida del discurso de las pacientes, sustituido, en parte, por el discurso de otros (familia, personal de cuidados, o discurso institucional) (García-Díaz, Jiménez-Lucena, 2010). Dentro de este juego clasificatorio de los diagnósticos kraepelinianos, se estableció una relación entre esas categorías o compartimentos, donde se trataba de ubicar a cada paciente, y las propias mujeres, dando lugar a discursos y prácticas dentro y fuera del manicomio. El empeño positivista de la psiquiatría se topó con dificultades para definir y cuantificar las conductas de las mujeres, por lo que fueron considerados como “datos objetivos” informaciones sobre las familias, enfermedades previas que pudieran justificar la “degeneración” de su psique, y lo que se suponían manifestaciones de la locura como las conductas, los hábitos, la higiene, el lenguaje, la forma de vestir, las relaciones personales, el cuerpo de las mujeres, las ideas religiosas y políticas (García-Díaz, 2019). La historia clínica, como lugar de construcción de la enfermedad mental, teje una matriz explicativa de la supuesta “anormalidad” de las pacientes, fundamentándola en el relato de vida de estas mujeres elaborado por terceros: familiares, vecinos y profesionales (Bedoya-Hernández, Castrillón-Aldana, 2018; García-Díaz, Jiménez-Lucena, 2010).

En ese intento de positivización en el Hospital Psiquiátrico Provincial de Málaga, se construyó un modelo de historia clínica que, en teoría, trataba de recoger información acerca del proceso mórbido de las pacientes. Frente a la alta frecuencia de datos sociodemográficos recogidos como la edad (97,4%), el estado civil (95%), lugar de nacimiento (88,5%), la profesión u ocupación es el dato personal menos recogido, tan solo un 30% de las historias lo especifican. Entre los documentos que sí lo recogieron las labores del hogar anotadas como “sus labores”, “labores propias de su sexo” o “su casa” aparecen en 198 mujeres (80% de los recogidos y el 24,4% del total); como sirvientas aparecen 25 mujeres y como costureras diez; el resto se reparten en otras ocupaciones como tres maestras, dos enfermeras, dos cocineras, una religiosa, una portera, una cigarrera y una trabajadora de fábrica (García-Díaz, 2019, p.147). Un aspecto relevante es el gran número de historias en las que no se cumplimentaban estos datos, lo que puede estar en relación con una estrategia de silenciamiento y negación básica en las prácticas de subalternización. La poca importancia que se daba a la recogida de estos datos en las historias de mujeres^16^ contrasta con el interés de las pacientes por hacer constar que se ocupaban del cuidado de otros, desarrollaban actividades fuera del hogar, como blanquear casas, planchar o coser, sin ningún tipo de relación contractual y con una baja remuneración que, sin embargo, constituían un soporte de la economía familiar (Sánchez, 2009, p.21). Es evidente que el que estos datos apareciesen en uno u otro lugar de la historia clínica tiene relevancia: la negación a hacer constar la profesión u ocupación en los datos sociodemográficos está configurando una determinada subjetividad que expulsa a las mujeres del mundo del trabajo. Con esto, se elabora un dato “objetivo” que va a servir en el proceso clasificatorio para definir la “normalidad” y la “anormalidad” de forma que, el que las mujeres realicen un trabajo remunerado, se constituiría en un hecho anómalo, y esto se va a utilizar para clasificar, patologizar.

Otros datos relacionados con el ingreso quedaban plasmados en el documento clínico: fechas de ingreso y alta, causa del alta y del fallecimiento si se producía en la institución, así como el médico que ordenaba el ingreso, recogido solo en el 44,8% de las historias.^17^ Este era el único lugar del documento donde figuraba el nombre de los facultativos que intervenían en todo el proceso, aunque las mujeres podían ser entrevistadas y tratadas por otros médicos de la institución. En relación a esto, resulta imposible conocer la identidad del psiquiatra que trataba a las mujeres a lo largo del proceso asistencial, ya que las anotaciones en la evolución no estaban firmadas. Se generaba así una abstracción sobre el experto, como si diera igual un profesional que otro, pretendiendo con la “expulsión del sujeto” un conocimiento libre de subjetividad, característica propia del pensamiento positivista (García-Díaz, Jiménez-Lucena, 2010, p.130).

**Figura 1:**
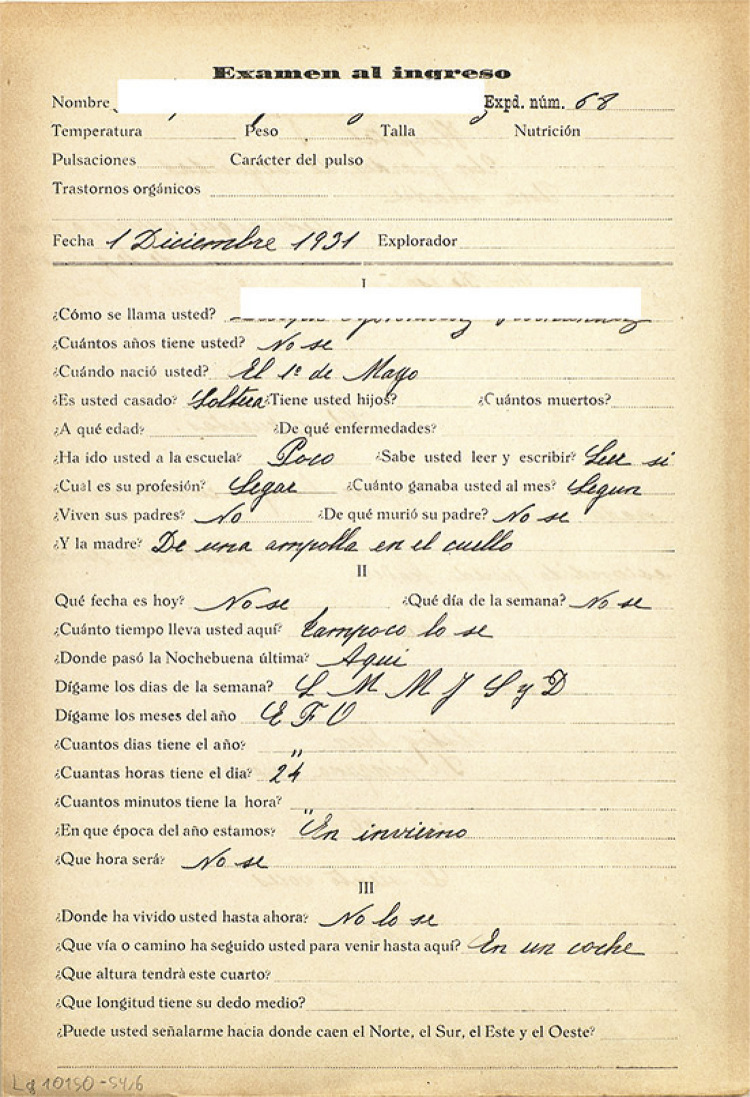
Examen al ingreso (Historia clínica…, 1931)

En la misma línea, se realizaba un cuestionario estructurado llamado “Examen al ingreso” (Figura 1) cuyos contenidos tenían que ver con operaciones matemáticas básicas y preguntas de lógica. Estos cuestionarios aparecen en las historias clínicas de la institución de mujeres entre 1927 y 1939 en un total de 23 casos. Es destacable señalar la dificultad de las pacientes para responder a la mayoría de las preguntas debido al bajo nivel educativo de la época.

Otras vías de positivización plasmadas en la historia clínica tenían que ver con la realización de pruebas diagnósticas como el test de Wasserman para detectar los casos de sífilis. Kraepelin señaló al descubrimiento de la espiroqueta como una de las contribuciones más importantes para mostrar el origen biológico de las enfermedades mentales. Proporcionaba una etiología positiva, demostrable, que podía ser aislada (Caponi, Martínez-Hernáez, 2013, p.471). El test fue aplicado en el 2,34% casos de las historias totales analizadas. Hay que resaltar cómo va disminuyendo el número de pruebas realizadas por periodos históricos, un 81% de las historias clínicas consultadas contienen este tipo de pruebas en el primer periodo analizado (1909-1936), un 63% durante la Guerra Civil y solo un 16% entre 1939 y 1950. Esta disminución del número de test durante los años 1940 puede tener un doble motivo: por un lado, la dificultad para conseguir los reactivos durante la posguerra, tanto por el aislamiento como por la situación económica; pero también pudiera estar provocada por una falta de reconocimiento de la sexualidad de las mujeres. La valoración de este asunto hace necesaria el análisis de la evolución cuantitativa de los test en las historias clínicas de hombres que nos proponemos abordar para ofrecer una respuesta más certera a esta cuestión.

**Figura 2:**
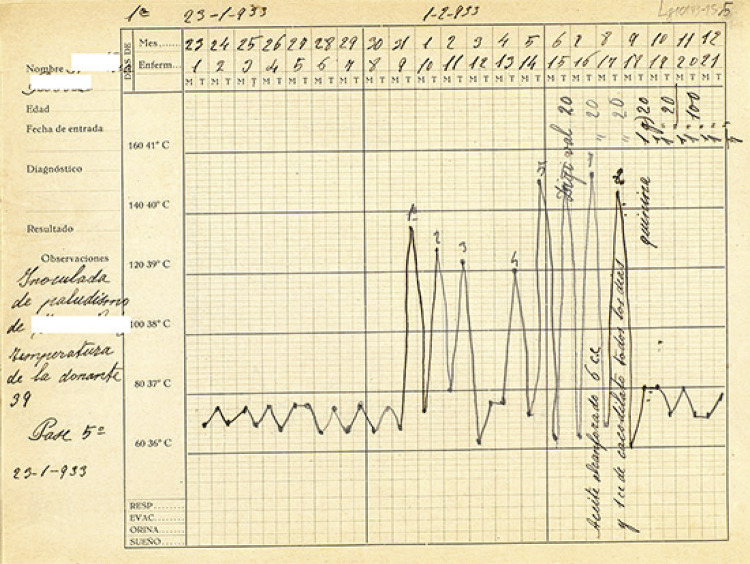
Gráfico de paciente tratada con malarioterapia (Historia clínica…, 1933)

**Figura 3:**
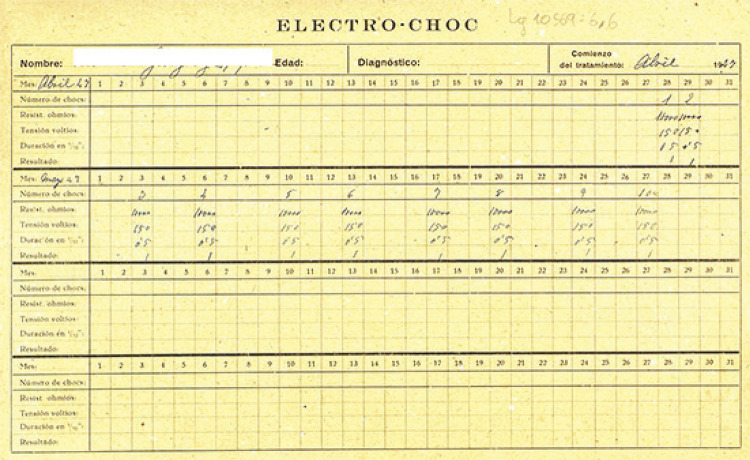
Hoja de recogida de aplicación de electrochoque (Historia clínica…, 1947)

Dentro de las estrategias de positivización también se incluyeron las gráficas de la aplicación de piretoterapia, inducida por diferentes vías tales como inyecciones subcutáneas de trementina o sulfatos, inoculación de vacunas anti tifoideas y anti estafilocócicas, y la malarioterapia (Figura 2), así como de los resultados de los tratamientos por choques (insulínico, cardiazólico y electrochoque). Este último comenzó a ser usado en el Manicomio Provincial de Málaga en 1941 (Figura 3) (García-Díaz, 2019, p.228).

El acto de diagnosticar ha sido señalado por la filosofía de la ciencia como uno de los mecanismos más usados por el discurso psiquiátrico (científico médico en general) para clasificar a grupos humanos y crear realidades desde el momento en el que a un conjunto de conductas se les aplica un nombre (Christiansen, 2009). Basándose en el modelo kraepeliniano, que fue hegemónico en la psiquiatría occidental en la primera mitad del siglo XX, la esquizofrenia fue la enfermedad más diagnosticada en la sala 20 del Manicomio de Málaga en la primera mitad del siglo XX (25% del total) (García-Díaz, 2019). Frente a los intentos de positivización del saber psiquiátrico, diferentes autores señalan que el alienismo del siglo XIX y principios del XX trató de introducir la subjetividad en el desarrollo de las disciplinas “psi” (Huertas, 2010). El diagnóstico de esquizofrenia fue problemático bajo esta visión positiva-subjetiva. Si bien se generalizó su uso desde la medicina positivista, permitiendo desarrollar tratamientos experimentales para su pretendida curación, el psicoanálisis también facilitó su comprensión desde su visión de ruptura del sujeto y el lenguaje (Álvarez, Colina Pérez, 2011, p.14; Novella, Huertas, 2010). A pesar de este interés, la realidad asistencial muestra la tendencia positivista de los profesionales en las fuentes analizadas. En las historias clínicas del manicomio malagueño se describía cómo las mujeres clasificadas bajo la etiqueta esquizofrenia resistían a las prácticas coercitivas, poniendo en marcha diferentes estrategias como fugas de la institución, negativas a recibir tratamientos y escritos de denuncia. Entre estas estrategias identificamos elementos de resistencia emocional que podemos reconstruir a partir de los relatos que componen la historia clínica. Esta presencia pone en evidencia la existencia de grietas por las que se introducían las subjetividades de las pacientes en la práctica psiquiátrica (García-Díaz, Jiménez-Lucena, 2010). Estas acciones que formarían parte de resistencias no estructuradas, en el sentido que apuntan Rosón y Medina Domenech (2017) para regímenes opresivos, nos hablan de una oposición a la dinámica totalizadora de la institución y la familia.

Así, la subjetividad de las pacientes de la sala 20 ha emergido en las historias clínicas de diferentes formas. Por un lado, como fuente directa, a través de los escritos producidos por ellas mismas durante el internamiento hemos podido conocer sus experiencias en la institución. Por otro lado, fuentes indirectas, como los relatos recogidos en las historias clínicas con entrecomillados, nos han permitido aproximarnos a esa subjetividad. Estas últimas han sido más frecuentes debido a los altos índices de analfabetismo de la población femenina (Ramos, 1993; Peinado Rodríguez, 2012), que ha hecho que, del total de 811 registros, solo en diez historias clínicas aparezcan cartas de mujeres (García-Díaz, 2019, p.137). Con unas y otras fuentes tratamos de reconstruir un discurso fragmentado y en parte silenciado por la institución ya que el etiquetado de las mujeres “ahogaba” sus relatos. Estos discursos señalaban a factores sociales, económicos, políticos, familiares, vivencias relacionadas con el cuerpo, la maternidad y la crianza de los hijos como causantes de su malestar.

En este sentido, cartas como las de L.M.S. muestran la importancia de la subjetividad en el diagnóstico psiquiátrico. La divergencia de los discursos de la paciente, la familia y los profesionales de la psiquiatría ponen de manifiesto el concurso de diferentes condicionantes políticos y sociales en el proceso asistencial. L.M.S. fue recluida en la institución psiquiátrica de Málaga en abril de 1938, cuando la ciudad acababa de ser tomada por el bando franquista durante la Guerra Civil Española. Se fugó a los nueve días de su ingreso volviendo a reingresar el 22 de mayo para volver a fugarse el 29 del mismo mes. Por último, ingresó el 1 de junio y fue dada de alta cinco días después. En su historial han sido encontradas varias cartas que escribió durante su internamiento. La primera la escribió en una servilleta, dirigida al señor gobernador civil, y al señor delegado de orden público donde denunciaba la situación en la que se encontraba: “Muy señores míos: Llevo quince días de muerte encerrada aquí perdiendo tres más de vida … necesito que, aunque solo sea un día más lo que yo debo estar aquí, me trasladen a un lugar conveniente donde siquiera pueda dormir. Esta petición a todas veces lícita me ha sido desatendida” (Historia clínica…, mayo 1938, p.177)*. *En su escrito hacía referencia a la situación durante el conflicto: “Hay innumerables refugiados que debieran haberse contentado con su suerte allá o contentándose con pisar tierra nacional sencillamente. Yo no necesito ni de uno ni de otros no necesito ser gravosa pues tengo disposición para desenvolverme en mi vida de manera honorable necesitando solo que no me entorpezcan y me den el pasaporte que solicito” (p.177).

La segunda carta, escrita esta vez en folio, es remitida a su hermano:

Querido hermano: … El tiempo pasa y quien yo creía que debiera habérmelo solucionado, que eran las autoridades de aquí, no se han tomado la menor molestia en hacerlo. No seré más explícita ni tú debes serlo. Solo te diré que me hayo envuelta en algo parecido a lo que a todos suele ocurrir en tiempos de guerra y que, en unos, como en mí, será pasajero y sin consecuencias; no así en otros que hasta se compromete su porvenir y su profesión. Por eso, aunque sea prestado que pidas, envía 100 pesetas y saldré de esta pesadilla. Ya escribiré más despacio y mientras recibe un abrazo de tu hermana (Historia clínica…, mayo 1938, p.177).

Según refería el hermano, L.M.S. fue atendida previamente por un psiquiatra en Madrid, que diagnosticó esquizofrenia y recomendó como tratamiento “distracción y educación de la voluntad” (Historia…, mayo 1938, p.177). De esta forma, las vivencias extremas o traumáticas se transformaban en síntomas que eran usados para justificar el ingreso de una mujer en un manicomio. Ella hacía alusión a contenidos políticos que pudieron estar detrás del ingreso a la institución, teniendo en cuenta que el primer ingreso se produjo en Málaga, dos meses después de la ocupación franquista de la ciudad. Aunque en su historia clínica no figura ningún diagnóstico ni tampoco tratamiento alguno, el discurso del hermano sobre la posibilidad de padecer una esquizofrenia justificó su permanencia en la institución. De la misma manera, fue la familia la que procuró su salida del manicomio en 1941.

Las dos cartas escritas por L.M.S. no fueron enviadas, ya que permanecieron dentro de su historial clínico, por tanto, se ejerció una censura sobre lo producido por esta paciente dentro de la institución.^18^ El hecho de que sus cartas quedaran en la historia clínica sin ser enviadas también nos indica que la subjetividad de la paciente no fue tenida en cuenta, y su discurso permaneció mudo para familiares e instituciones, invisibilizándose su deseo de autonomía y su capacidad para tomar sus propias decisiones.

Los datos recogidos sobre otra paciente diagnosticada de esquizofrenia ingresada en septiembre de 1936, año del levantamiento militar que provocó la Guerra Civil, incidían en la divergencia de las subjetividades y su resultado en los procedimientos psiquiátricos. El psiquiatra dio relevancia al hecho de que “al entrar pide permiso para sentarse haciéndolo en la silla que le decimos que no. Se niega a contestar, manierismos, gesticulante, mutista. En el departamento se rompe la ropa, gustándole estar siempre en pantalones, al parecer tendencia homosexual” (Historia clínica…, 1936, p.78). Así, se ponía el énfasis en las conductas de desobediencia y resistencia de la paciente, estableciéndose una relación entre la orientación sexual y su vestimenta. Otros fueron los términos en los que el marido describió la experiencia de la paciente. Su relato fue recogido por el psiquiatra de la siguiente forma:

No existen enfermedades mentales ni tampoco ella ha dado muestras de trastorno psíquico. Atribuye el marido su enfermedad a los episodios convulsivos tan violentos de la presente revolución. Vivían en San Roque con seis hijos; cuando entraron las fuerzas militares huyeron dejando a los hijos con los abuelos, entonces empezaron los trastornos mentales, el marido la llevó en peregrinación de pueblo en pueblo: Manilva, Casares, Estepona, deteniéndose algunos días, pero en este último punto el estado psíquico empezó de tal manera, con alteraciones motoras y de la afectividad, que hubo de ser trasladada a este manicomio (Historia clínica…, 1936, p.78).

En el discurso del marido se puso más el acento en la vivencia de separación de sus hijos, y la fragmentación de su identidad por las vivencias traumáticas de la guerra; sin embargo, el contenido que recogió el psiquiatra hacía referencia a los síntomas conductuales y a la orientación sexual de la paciente. Esta prioridad de lo sexual frente a lo traumático de la guerra puede estar en relación con la idea que planteaba otro psiquiatra afín al régimen franquista, Sarró Burbano, que, si bien por un lado reconocía la importancia de los factores exógenos en el desarrollo de las crisis psicóticas, jerarquizó estas causas entendiendo las de origen sexual y religioso las más potencialmente desestabilizadoras, frente a la mínima o nula importancia que tendrían los traumas de guerra en su interpretación (Dualde Beltrán, 2004, p.3580). De esta forma se negaba la experiencia traumática que la guerra provocaba en las personas que sufrían las alteraciones mentales y en quienes las acompañaban.

La psicopatía también fue un diagnóstico problemático en la institución. Cesar Lombroso, en los primeros años del siglo XX, aplicó la expresión “locura moral”, descrito por J.C. Pritchard en 1835, a mujeres prostitutas, cuyos síntomas serían la falta de afectos naturales hacia sus familiares, el rechazo de la maternidad, el alcoholismo, la codicia, la falta de pudor, ociosidad, vanidad, mentira y ligereza, así como la tendencia al juego (Lombroso, Ferrero, 1893). La sexualidad femenina estaba íntimamente ligada a la reproducción, por lo que, en estas mujeres se señalaba la separación entre sexualidad y maternidad. Así, las mujeres que no entendían su sexualidad desde la perspectiva de la maternidad podían ser susceptibles de diagnósticos como estos, locas morales en el siglo XIX y psicópatas ya entrado el siglo XX (Narvalaz, Jardón, 2010). En España, como ya hemos señalado, las mujeres que durante el franquismo fueron vinculadas a la defensa del régimen republicano eran clasificadas como psicópatas.^19^ A esto respondieron algunos de los ingresos realizados en el Manicomio Provincial de Málaga, como fue el caso de DBJ, de 54 años, ingresada en la institución en septiembre de 1940. En el apartado “antecedentes” de su historial, no se reflejó ningún dato acerca de ella ni de sus familiares, solo se señalaba “A disposición del psiquiatra militar”. Según se anotó en la historia, se resolvió un fallo del Tribunal de Justicia Militar de Tetuán, Marruecos, por padecer una reacción de tipo paranoide. Lejos de ser diagnosticada como paranoide, se le aplicó el diagnóstico de psicopatía durante su ingreso en la institución de Málaga. En el resto de la historia, se hacía referencia a su forma de estar en el psiquiátrico:

Cuidada, correcta, tranquila. Cose. A veces habla misteriosamente con otras enfermas y con ironía. Orientada. Se queja de estar incómoda en su familia pues no tiene correspondencia. Explica que por haber subarrendado en Tetuán una habitación a un señor y como le dijo que le hacía falta, la denunció como persona no adicta al movimiento. No cree que hayan juzgado su causa. No quiere explicar otras cosas y se las reserva por ‘no ser usted mi juez, si no mi médico y no es preciso’ (Historia clínica…, 1940, p.39).

Este fragmento nos muestra alguna de las estrategias de resistencia de las mujeres ingresadas como el silencio, la negativa a hablar por las consecuencias que pudieran tener sus declaraciones en un régimen dictatorial en el que se estaba desarrollando una práctica médica que, como hemos visto, psiquiatrizó idearios políticos. Además, la paciente hizo una clara definición de los espacios de dominación: el judicial y el médico, señalando que no tenía por qué contarle su problema judicial a un médico. Esta paciente acabo fugándose de la institución, en diciembre de 1941, formando parte de los 35 casos de mujeres que optaron por la estrategia de fuga, del total de historias analizadas.

Como hemos señalado con anterioridad, uno de los objetivos del estudio que Antonio Vallejo Nágera realizó en la cárcel de mujeres de Málaga, fue detectar la proporción de inferiores mentales en las filas marxistas. El retraso mental, a lo largo de la historiografía psiquiátrica, ha recibido diversas nomenclaturas en función de la escuela nosológica que predominara. Así, durante el siglo XIX, la escuela de alienistas franceses acuñó términos como idiocia, imbecilidad y debilidad mental. Basándose en la degeneración de la raza, Vallejo Nágera también conectó la idea de la inferioridad mental con la ideología marxista, argumentando que “el simplismo del ideario marxista y la igualdad social que propugna favorecen su asimilación por los inferiores mentales y deficientes culturales, incapaces de ideales espirituales, que hallan en los bienes materiales que ofrecen el comunismo y la democracia la satisfacción de sus apetencias animales” (citado en Huertas, 1996, p.116)*. *Sin duda la historia de A.V.G. respondía a esta elaboración ideológica de la psiquiatría franquista. Esta mujer de 25 años ingresó en el manicomio en junio de 1939; hasta ese momento había estado presa en la cárcel de mujeres de Málaga. Su historia clínica coincide con otras muchas en las escasas anotaciones que contiene; el psiquiatra que la atendió al ingreso, sin hacer constar su nombre, describió lo siguiente:

Entra correcta. Viene mal vestida. Llora y refiere que siempre la han tratado mal; los chicos la apedreaban … se anima cuando habla de su matrimonio con un francés de las Brigadas Internacionales que conoció en Barcelona: ha estado con él en Barcelona, y se tuvo que separar por ser cocainómano. Habla francés: conoce las expresiones francesas y el argot … La han traído de la Aduana; iba por la calle con unos pantalones y le pegaron y entonces fue detenida (Historia clínica…, 1939, p.98).

Con estos escasos datos, el psiquiatra dudó del diagnóstico ya que aparece tachada la palabra “psicopatía” en su historial. Finalmente, la decisión diagnóstica fue otra, igualmente ligada por la psiquiatría del momento a las personas relacionadas con la defensa de la Segunda República, la oligofrenia. Sin embargo, esta mujer sabía leer, escribir y hablaba francés, lo cual no era común en la época, dado el elevado índice de analfabetismo existente como ya hemos señalado. Su relación con un brigadista y su situación de presa influyó más en el diagnóstico que un dudoso déficit cognitivo del que no solo no hay datos en el documento clínico, sino que éstos parecen contradecirlo. También hay que destacar que nuevamente aparecen los pantalones como un elemento relevante para el psiquiatra, igual que en el caso de A.L.V. En ambas ocasiones fueron considerados como señal de desobediencia a la indumentaria típica femenina y asociados con “anormalidades”; en este caso se destacaba una prenda sobre la que se construyó el estereotipo de la miliciana durante la Guerra Civil lo que aquí se convertiría en un dato para el diagnóstico. A pesar de no existir ningún signo de patología mental en su historia clínica, a la paciente se le aplicó malarioterapia. Al igual que en el caso de D.B.J., la paciente se fugó del manicomio, en este caso en dos ocasiones entre 1939 y 1940.

## Consideraciones finales

El dispositivo manicomial diseñado en la provincia de Málaga participó de las ideas psiquiátricas hegemónicas globales que trataban de legitimar a la psiquiatría como ciencia positiva dentro de la medicina de laboratorio. Sin embargo, los intentos de positivización de los relatos de las pacientes dejan entrever las experiencias traumáticas vividas y, por tanto, los elementos de subjetivación dentro de la institución manicomial. El hecho de que los propios psiquiatras dudaran de establecer unos diagnósticos u otros, como en los casos planteados, apunta hacia la objetividad como una ilusión de la ciencia psiquiátrica difícil de conseguir, y pone de manifiesto la consideración de elementos subjetivantes que inclinaran la balanza hacia un diagnóstico u otro.

Los discursos y prácticas psiquiátricas estuvieron muy relacionadas con el contexto socio político español de la primera mitad del siglo XX, lo que se puede observar de forma evidente en los relatos recogidos de las pacientes durante la Guerra Civil y el primer Franquismo, en los que una pretendida “ciencia psiquiátrica” servía al régimen franquista para identificar y segregar a las mujeres defensoras de la República, con la idea de establecer un modelo de feminidad afín a la ideología totalitaria del nacionalcatolicismo, clasificando como “locas” a las mujeres que no respondieran a ese modelo. Durante el primer franquismo, la ideología del nacionalcatolicismo y la aversión a las mujeres republicanas, llevaron a etiquetar a las mujeres disidentes, bien por su vestimenta transgresora que relacionaban con una sexualidad considerada aberrante, bien por su ideología, como oligofrénicas, psicópatas y esquizofrénicas.

Por otro lado, como ha quedado recogido en los relatos clínicos, las mujeres implementaron estrategias de resistencia dentro del escaso margen del que disponían, siendo agentes activos en el devenir del manicomio. La pretensión de agencia por parte de las mujeres y las dificultades para conseguirla se evidencian en los relatos de los psiquiatras, pero sobre todo en las cartas escritas por las pacientes que en ocasiones quedaron sin ser enviadas a sus destinatarios. Los discursos de las mujeres internadas no eran escuchados ni eran considerados verdaderos, dándose prioridad a los discursos de familiares e institución. Sin embargo, esa intención de censura y negación de agencia por parte del dispositivo psiquiátrico ha posibilitado la conservación en un archivo histórico de una documentación que ha permitido el acceso a unas fuentes esenciales para la visibilización de las resistencias.

## NOTAS

1 Un discurso científico misógino se gestó desde la medicina para contrarrestar el cambio social que la lucha de las mujeres planteaba. Obras como las de Moebius, Nordau y Roberto Novoa Santos en nuestro entorno apoyaban la incapacidad intelectual de las mujeres para desarrollar trabajos en la esfera de lo público y acceder a la educación superior tratando de mantener la lógica público/privado. Para profundizar en esta cuestión, ver Ortiz (invierno 1996-1997).

2 Para profundizar en aspectos organizativos y asistenciales de la sala de mujeres de esta institución, ver García-Díaz (2018, 2020).

3 Presentamos porcentajes del estudio cuantitativo sobre el total de historias analizadas y contenidos de historias seleccionadas por su riqueza discursiva.

4 El Krausismo defendía la libertad de cátedra y la tolerancia académica frente al dogmatismo. En España, dio lugar a la Institución Libre de Enseñanza.

5 Para Novoa Santos (1908), las “mujeres originales” son las que se desmarcan del ideal femenino hegemónico, y plantean aspiraciones intelectuales y de formación, asimilando que estas mujeres tienen psique de hombre: “El juicio popular no anda descarriado cuando califica a estas mujeres de marimachos” (p.117).

6 Publicación periódica fundada en 1903 y editada en el Manicomio de San Baudilio de Llobregat, Barcelona.

7 Los estados premenstruales fueron usados durante las primeras décadas del XX para justificar políticas de género, como la incapacidad de las mujeres para tomar una decisión al votar o la atribución de responsabilidad jurídica en procesos de separación o divorcio.

8 María Cambrils fue una feminista socialista española, nacida en Valencia, que defendió un feminismo igualitario con una importante conciencia de clase y basado en la educación de las mujeres como elemento básico en su emancipación (Aguado, 2003).

9 Para un análisis sobre la historia del psicoanálisis en España, ver Lévy Lazcano (2019).

10 Juarros participó activamente en la cruzada científico-ideológica contra un feminismo “masculinizante” señalando que la aspiración de estas mujeres al acceso a las pautas sociales y educativas de los hombres serían perjudiciales y se oponía a su realidad biológica (Aresti, 2001, p.237).

11 La cuestión de la educación sexual en las mujeres desde el discurso eugenista tiene matices que hay que señalar. Las niñas, como futuras mujeres, eran depositarias de una carga ideológica sobre la importancia de su papel no solo como madres, sino como esposas y educadoras en el ámbito de lo privado, siendo, en ocasiones, las responsables de la degeneración y la enfermedad de los hijos. Para profundizar en esta cuestión, ver Diego Pérez y González Fernández (2014).

12 Recientes estudios sobre la psiquiatría franquista señalan que hubo un profundo desinterés del régimen por la atención psiquiátrica (Campos, González de Pablo, 2016). Sin embargo, queremos apuntar la siguiente idea: si bien los discursos y las prácticas institucionales mostraron parte de ese desinterés, en el ámbito de la relación psiquiatra-paciente-familia, la ideología nacionalcatólica y su idea de feminidad hegemónica psiquiatrizaron conductas y formas de vida que consideraron disidentes. Por otro lado, ese desinterés contrasta con el experimento sobre las mujeres “rojas” de la cárcel de la ciudad, trabajo llevado a cabo por Vallejo Nágera, en 1938, realizado en la Málaga ocupada (ver Huertas, 1996). Planteamos, pues, que es el abordaje microhistórico de las fuentes el que arrojaría más luz sobre estas dinámicas.

13 Psiquiatra y consejero Nacional de Educación y Sanidad durante el franquismo. Sus ideas fascistas iban unidas a una intensa misoginia que reflejó en sus investigaciones señalando la existencia de una “delincuencia marxista femenina” (Huertas, 1996, p.114). Para un análisis más profundo en torno a continuidades y discontinuidades sobre la psiquiatrización de comportamientos políticos en España, ver Campos (2021).

14 Sarah Wise aborda la cuestión de la psiquiatrización de las personas inadecuadas e inapropiadas en su trabajo de 2012, *Inconvenient people*.

15 Nos referimos al conjunto de prácticas discursivas y no discursivas encaminadas a la detección y expresión de datos cuantitativos, gráficas y mediciones que se pusieron al servicio de una lectura del acto psiquiátrico como puramente objetivo. Por otro lado, también nos referimos al espacio discursivo recortado, que no totalmente invisibilizado, de las mujeres-locas en el relato clínico. Como señala Fantin (2005, p.473) cuando se plantea el abordaje epistemológico del positivismo, se cuestionan aspectos ontológicos y conceptos centrales en la ciencia como realidad, verdad, hecho, teoría y supuestos, entre otros, conceptos sobre los que pivotaban teorías y clasificaciones diagnósticas.

16 Estamos realizando el estudio de las historias clínicas de pacientes hombres en la misma institución; en este caso, el porcentaje de las historias revisadas (200 historias de 1945 y 1946) hacen constar la profesión en un 52%.

17 En el periodo estudiado los criterios de ingreso/alta fueron modificados tras el decreto de 1931, otorgando más protagonismo al criterio médico frente al Real Decreto de 19 de mayo de 1885. Ver Decreto... (jul. 1931).

18 En Villasante (2018) se ha analizado la cuestión de la correspondencia de pacientes en el Manicomio de Santa Isabel de Leganés, señalándose que se ejerció una censura sobre las cartas no enviadas por temor a que en el exterior se conocieran las condiciones de vida de los internos.

19 La cuestión de la feminidad durante el franquismo estuvo regulada por instituciones como el Patronato de Protección de la Mujer. Aunque existía desde 1902, sus funciones y atribuciones fueron cambiantes a lo largo de la primera mitad del siglo XX, dependiendo del signo político que estuviera en el poder. Con el franquismo, tuvo una clara intención de recluir y reeducar a jóvenes de conductas consideradas inmorales (Bandrés, Zübieta, Llanova, 2014; Guillém Lorente, 2018).
